# Outcomes of Invasive Fungal Infections Treated with Isavuconazole: A Retrospective Review

**DOI:** 10.3390/pathogens13100886

**Published:** 2024-10-11

**Authors:** Vanessa Gow-Lee, Omar M. Abu Saleh, Courtney E. Harris, Jennifer J. Gile, Nadia Akhiyat, Supavit Chesdachai

**Affiliations:** 1 Division of Infectious Diseases and Geographic Medicine, Stanford University, Palo Alto, CA 94305, USA; 2 Division of Public Health, Infectious Diseases, and Occupational Medicine, Department of Medicine, Mayo Clinic, Rochester, MN 55905, USA; abusaleh.omar@mayo.edu (O.M.A.S.); chesdachai.supavit@mayo.edu (S.C.); 3 Division of Infectious Diseases, Medical University of South Carolina, Charleston, SC 29425, USA; harricou@musc.edu; 4 Department of Oncology, Mayo Clinic, Rochester, MN 55905, USA; gile.jennifer@mayo.edu; 5 Department of Cardiovascular Medicine, Mayo Clinic, Rochester, MN 55905, USA; akhiyat.nadia@mayo.edu

**Keywords:** central nervous system infections, invasive fungal infections, isavuconazole, salvage therapy, step-down therapy

## Abstract

Background: Isavuconazole (ISA) has a favorable side effect profile that makes it attractive for treatment of invasive fungal infections (IFI). It carries FDA approval for invasive aspergillosis and mucormycosis, but there are fewer data for other organisms and non-pulmonary infections. We conducted this review to investigate how ISA performed at treating IFI, with an especial interest in these non-approved indications. Methods: We retrospectively identified and reviewed 131 patients who received ISA as treatment for IFI at our institution, some of whom received ISA as their first anti-fungal therapy and others who received ISA as either step-down therapy or salvage therapy. We identified the microbiologic cause of infection as well as the anatomic site involved for each patient. We then classified patients according to their response to ISA: namely cured, partially responded, or stabilized. Results: The majority of patients were immunocompromised (*n* = 76, 58%). ISA was used primarily as a secondary therapy (*n* = 116, 89%); either as a step-down/switching from other agents, or as salvage therapy. The most common reasons for switching to ISA were toxicities with prior agents followed by QT prolongation. Although pulmonary aspergillosis and mucormycosis were represented in more than half of the cohort, ISA was also used off-label for treatment of other organisms such as endemic fungi (*n* = 19, 15%) as well as central nervous system (CNS) infections (*n* = 15, 11%). We have described the detailed clinical characteristics of these CNS infections cases. The overall clinical response rate varied by type of infection and site involved (57–73% response rate). Conclusions: We demonstrated encouraging clinical responses, particularly outside the FDA-approved indications, as well as good tolerability. This report highlights the critical need for expanded scope of prospective studies to delineate the efficacy of this better-tolerated agent, especially in central nervous system infections.

## 1. Introduction

Invasive fungal infections (IFI) implicate multiple organ systems and carry high morbidity and mortality. Patients who develop invasive fungal infections often have an underlying immunocompromising condition. Specific risk factors include a high use of immunosuppressing drugs, greater immunologic disparity between transplant donor and recipient (e.g., human leukocyte antigen mismatch), prolonged neutropenia, prior IFI, and comorbidities such as renal failure after transplantation [1,2,3]. The incidence of IFI is roughly 4% in bone marrow transplant (BMT) recipients and roughly 2% in solid organ transplant (SOT) recipients, although the incidence varies widely with different types of organs (e.g., up to 12% in small bowel transplantation and as low as 1.3% with kidney transplantation) [4,5]. Similarly, outcomes vary by type of infection and by patient population, with BMT recipients typically having worse outcomes than SOT recipients. The 1-year survival rate has been reported as 25–33% in BMT and 59–73% in SOT recipients [4,5].

Treatment of invasive fungal infections is also often fraught with complications. Given vulnerable patients are often on many other medications, such as chemotherapy, drug-drug interactions are common when a fungal infection is treated with triazoles, which are inhibitors of various cytochrome isozymes [6,7]. Tolerability is also a frequent challenge with antifungal agents, with adverse events including renal dysfunction, liver toxicity, and QTc prolongation. Infections in non-pulmonary sites can also have unique challenges, with the central nervous system (CNS) being a prototypical site. There are limited anti-fungals that can achieve therapeutic levels in the CNS space, and historically CNS infections have been treated primarily with amphotericin, fluconazole, and voriconazole [8].

In light of these issues, the relatively novel triazole isavuconazole (ISA) offers many advantages. ISA’s oral bioavailability is >97%, its pharmacokinetics are linear and more predictable than that of other azoles, and although drug interactions can still occur, the potential is lower with ISA compared with other azoles [9,10,11]. It has a wide volume of distribution, and based on animal studies, appears to penetrate the blood-brain barrier and access the CNS [12,13]. Its spectrum of activity includes both yeasts (including *Candida* species [spp.] and *Cryptococcus* spp.) as well as molds (including *Aspergillus*, dematiaceous molds, and certain species of zygomycetes) [9,12].

The data for ISA are most robust for primary invasive aspergillosis or mucormycosis, with most cases in the relevant trials involving pulmonary infections [14,15,16]. These trials have led to FDA approval for ISA for treatment of invasive aspergillosis and mucormycosis. However, the scope of ISA use remains poorly defined beyond these clinical scenarios, especially for non-pulmonary infections [17]. A few small studies have examined the efficacy of off-label use of ISA in infections such as *Cryptococcus* and dimorphic fungi, as well as in CNS infections, and suggest encouraging outcomes [18,19,20,21]. Given these limited data, we conducted this study to identify patterns of use of ISA at our institution, the types of infections for which ISA was used, and what the outcomes were in treating IFI, with an especial interest in off-label use.

## 2. Materials and Methods

We retrospectively reviewed all patients with IFI who had been treated with ISA at the Mayo Clinic Hospitals (Minnesota, Arizona, and Florida) between 1 January 2010, and 30 March 2020. These patients were diagnosed with IFI by an infectious diseases specialist, and ISA was selected based on the clinical discretion of treating providers. Inclusion criteria included (1) having had a clinical diagnosis of definite or probable IFI as based on the 2019 European Organization for Research and Treatment of Cancer and the Mycoses, and (2) having received ISA as a treatment agent for their IFI [22]. Patients receiving ISA as a prophylactic agent were excluded. The study was approved by the Mayo Clinic institutional review board. Patients were identified via an enterprise-wide patient database. Individual patients’ clinical data were reviewed and collected using electronic medical records and Research Electronic Data Capture (REDCap) tools.

We collected variables on (1) the role of ISA (as primary, salvage, or step-down therapy), (2) the reasons clinicians chose to start or stop ISA, and (3) the length of treatment. We also reviewed the sites of infection, microbiologic diagnoses, overall mycologic responses, and survival at six weeks after ISA initiation. Demographic data obtained included clinical history, transplant status, and immune status.

Neutropenia was defined as absolute neutrophil count <1500 cells/µL. We defined ISA treatment as primary therapy when ISA was the first antifungal used; salvage therapy when ISA was used after another azole failed or was contraindicated; and step-down therapy when ISA was the first oral agent used after intravenous antifungal induction therapy. The mycologic response was determined based on the patient’s clinical status, imaging studies, and microbiological data (e.g., clearance of blood cultures or decrease in *Coccidioides* complement fixation titers). Response to treatment was defined as complete resolution (no further evidence of infection), partial resolution (infection present but significant improvement), or stabilization of infection (infection present but not progressing).

Continuous variables were reported as medians with an interquartile range (IQR), whereas categorical variables were expressed as count (*n*) and percentage (%). All statistical analyses were performed using BlueSky Statistics version 7.40 (Chicago, IL, USA).

## 3. Results

We identified 131 cases of ISA treatment for IFI, with 37% females. The median age of the patients was 62 years (IQR: 48–70). A significant majority of our study subjects were ethnically white (81%). Given that the largest hospital among our study sites is located in southeastern Minnesota, this ethnic majority reflects the local demographics of our catchment area. Of all patients, 44% had an active malignancy and 32% had had a prior transplant. Baseline demographics are detailed in Table 1.

ISA was dosed at 372 mg daily and was given either orally or intravenously based on the clinicians’ decisions. ISA was used as primary therapy in 11%, step-down therapy in 26%, and salvage therapy in 63% of cases. The reasons that antifungal therapy was switched to ISA were toxicities with other agents (29%), QTc prolongation (19%), failure of preceding therapy (17%), drug-drug interactions (10%), a favorable susceptibility pattern (9%), inadequate drug levels of primary therapy (3%), cost of primary therapy (1%) or no clear reason (11%). The median duration of ISA use was 60 days (IQR: 11–274 days), with 23% of patients receiving ISA for over six months.

Although most cases involved pulmonary infections and the treatment of *Aspergillus* or *Mucorales*, there were also off-label uses. Table 2 illustrates the distribution of infection sites, the cause of infections, and the response rates to ISA. Given the difficulty of treating fungal infections in the CNS and the paucity of data on ISA in these scenarios, we detailed our 15 CNS infection cases separately in Table 3. Of the CNS cases, ISA was used as salvage therapy in 12 (80%) cases. Six cases (40% of the CNS cases) had resolution, three (20%) had a partial response, three (20%) had stabilization, and three (20%) had a progression of the infection.

Treating clinicians discontinued ISA early in 79 (62%) patients. Reasons for early discontinuation included death or comfort care (33%), intolerance (22%, primarily gastroenteritis and rashes), failure of ISA (22%), cost or access (7%), drug-drug interaction (4%), issues with absorption or drug delivery (4%), microbe susceptibility (1%) and other reasons (8%). No significant adverse effects were noted in 30 cases of long-term ISA use (i.e., >six months).

## 4. Discussion

In this retrospective review, the response to ISA varied by site of organ involvement, with an up to 73% response rate for invasive fungal sinusitis and as low as 57% for cases of fungemia. ISA also had varying response rates for different microbial infections; in cases of aspergillosis and mucormycosis (the infections for which ISA carries FDA approval), there were clinical responses in 67% and 53% of cases, respectively. In comparison, 72% of yeast (predominantly *Candida* infections), 65% of dimorphic fungi, and 86% of dematiaceous mold infections had a response to ISA. About half of the cases were for off-label indications.

Given that the available data on ISA efficacy are predominantly in pulmonary infections, we were especially interested in our invasive CNS fungal infection cases. There are few antifungal agents with reliable CNS penetration, and testing of the drug penetration is limited by both poor data in human subjects and complicated pharmacokinetics and distribution, making animal studies challenging to interpret [23]. The primary agent typically used is liposomal amphotericin B, which has significant adverse effects. Voriconazole is the azole with the most clinical data in CNS infections, but it also has adverse effects including hallucinations, photosensitivity, cutaneous malignancies, and periostitis [23,24,25]. Because of these undesirable side effect profiles, reliable data on the role of ISA in CNS infections is highly desirable but lacking. There have been a few other observational studies of ISA use for CNS infections, including coccidiomycosis, paracoccidiomycosis, histoplasmosis, and cryptococcosis [18,19]. These studies showed mixed results, but case numbers were limited to <10 patients for each microbial etiology. In our 15 cases of CNS infections, we found that ISA had a 73% response rate in primarily salvage therapy cases, indicating some promise; however, this finding was divided between ≥6 microbial etiologies.

This report has important limitations. As a retrospective study, there is the potential for selection bias in which patients’ clinicians decided to treat with ISA. For example, given the high use of ISA as salvage therapy (when other anti-fungal therapy was judged to be insufficient), this patient cohort may have had comparatively more severe and recalcitrant fungal infections. This could have portended a higher-than-average likelihood of a poor outcome, regardless of which anti-fungal they received, and could have led to judged failure of ISA therapy. Conversely, any potential benefit of ISA therapy in our study subjects may have been confounded by a partial response to the initial anti-fungal therapy.

Another important limitation is the heterogeneity in infection sites and microbial diagnoses among our patient cohort, which inherently leads to a smaller sample size for each type of infection. This limits interpretation of ISA use for individual indications. Unfortunately, this is reflective of the general paucity of data that currently exists in the literature.

And finally, as a retrospective review, this article’s scope did not include any comparator groups who received other antifungals. Although our response rate to ISA seems to approximate response rates with other anti-fungal therapies as reported in the large TRANSNET cohorts of invasive fungal infection, this review was not a direct comparison between ISA and other drugs and so should be interpreted with caution [4,5].

## 5. Conclusions

ISA is a broad-spectrum antifungal agent with multiple attractive attributes, yet it remains underutilized. The concerns about breakthrough infections despite ISA prophylaxis and the lack of high-quality data for other indications besides the treatment for aspergillosis and mucormycosis likely contribute to this trend [26].

Our cohort of patients with IFI were treated with ISA which included both on- and off-label uses. Of particular interest were the cautiously encouraging responses in microbial etiologies and organ infections that are less well-represented in the literature thus far, especially CNS infections. Given the significant advantages of ISA and the high morbidity and mortality posed by invasive fungal infections, establishing when this better-tolerated agent can be reliably used is of great clinical consequence. Prospective trials comparing ISA to other known agents for a broader set of indications are needed to establish the role of ISA.

## Figures and Tables

**Table 1:** Baseline Demographics between patients who received ISA as primary vs. secondary therapy.

	Total 131 Patients (*n*: 131)	ISA Used as Primary Agent (*n*: 15)	ISA Used as Secondary Agent * (*n*: 116)
Median age (interquartile range)	62 years old (48–70)	63 years old (55–73)	61 years old (48–69)
Gender (%)			
Male	83 (63%)	10 (67%)	73 (63%)
Female	48 (37%)	5 (33%)	43 (37%)
Race (%)			
White	106 (81%)	13 (87%)	93 (80%)
Black	6 (5%)	1 (7%)	5 (4%)
Hispanic	2 (2%)	0 (0%)	2 (2%)
Asian	1 (1%)	0 (0%)	1 (1%)
Native American	2 (2%)	0 (0%)	2 (2%)
Other or Unknown	14 (11%)	1 (7%)	13 (11%)
Active Malignancy (%)	58 (44%)	9 (60%)	49 (42%)
Hematologic	48 (83%)	7 (78%)	41 (84%)
Solid	10 (17%)	2 (22%)	8 (16%)
Prior Transplant (%)	42 (32%)	5 (33%)	37 (32%)
Bone Marrow	21 (50%)	2 (40%)	19 (51%)
Solid Organ	21 (50%)	3 (60%)	18 (49%)
Immunosuppression at IFI diagnosis (%) ^Δ^	76 (58%)	10 (67%)	66 (57%)
Neutropenia ^δ^	30 (40%)	1 (10%)	29 (44%)
Chemotherapy	40 (53%)	7 (70%)	33 (50%)
Transplant Medications ^σ^	47 (62%)	8 (80%)	39 (59%)
Prednisone > 20 mg daily	23 (30%)	3 (30%)	20 (30%)

* Secondary agent includes both salvage and step-down therapy. ^Δ^ Patients could have multiple types of immunosuppression, so the rates of individual causes sum up to >100%. ^δ^ Neutropenia was defined as an absolute neutrophil count (ANC) <1500 cells/microliter. ^σ^ Transplant medications included tacrolimus, sirolimus, and mycophenolate mofetil.

**Table 2:** Outcomes of isavuconazole use for invasive fungal infections treatment grouped by organ involvement and microbial etiology.

	Number of Patients (% of Total 131 Cases)	ISA Used as Secondary Therapy (% of Each Category)	Median Duration of ISA Use in Days (Interquartile Range)	Mortality at 6 Weeks *	Response Rate to ISA ^Δ^
Organ Infection ^δ^					
Pulmonary	78 (60%)	65 (83%)	59 (9–259)	15%	65%
Sinus	16 (12%)	16 (100%)	53 (25–174)	13%	63%
Central nervous system	15 (11%)	15 (100%)	407 (152–570)	0%	73%
Bloodstream	7 (5%)	7 (100%)	10 (7–11)	29%	57%
Microbial Infection ^δ^					
*Aspergillus* spp.	51 (39%)	44 (86%)	67 (16–246)	18%	67%
*Candida* spp.	20 (15%)	20 (100%)	13 (10–64)	20%	70%
*Coccidioides* spp.	15 (11%)	13 (87%)	241 (17–580)	0%	73%
*Mucormycosis*	15 (11%)	13 (87%)	36 (21–122)	20%	53%
*Dematiaceous molds*	7 (5%)	5 (71%)	368 (67–590)	0%	86%
*Histoplasma* spp.	4 (3%)	4 (100%)	19 (7–56)	0%	50%
*Cryptococcus* spp.	3 (2%)	3 (100%)	303 (156–496)	0%	100%

* Six weeks from the time of ISA initiation. ^δ^ Patients could have multiple sites of infection/organisms and not all the sites/organisms were illustrated in this table. ^Δ^ Response to ISA judged at the end of therapy, including complete resolution, partial resolution, and stability.

**Table 3:** Cases of invasive fungal infection involving the central nervous system that were treated with isavuconazole.

Patient	Clinical Syndrome	Microbial Etiology	Use of ISA	Antifungal Used before ISA	Duration of ISA in Days	Clinical Outcome	Death at 6 Weeks *
1	Cerebral mucormycosis	*Apophysomyces* spp.	Step-down therapy	Amphotericin	22	Progression	No
2	Skull base osteomyelitis	*Aspergillus* spp.	Salvage	Amphotericin, Posaconazole	568	Stabilization	No
3	Meningitis	*Aspergillus fumigatus*	Salvage	Voriconazole	37	Progression	No
4	Skull base osteomyelitis	*Aspergillus fumigatus*	Salvage	Voriconazole	407	Stabilization	No
5	Cerebral abscess	*Aspergillus fumigatus, Aspergillus flavus*	Salvage	Amphotericin, Voriconazole, & Posaconazole	1035	Resolution	No
6	Epidural abscess	*Aspergillus fumigatus*	Salvage	Voriconazole	103	Progression	No
7	Cerebral abscess	*Caldophialophora bantiana*	Salvage	Voriconazole	573	Resolution	No
8	Meningitis	Coccidioides	Salvage	Fluconazole & Voriconazole	Unclear	Stabilization	No
9	Meningitis	Coccidioides	Salvage	Amphotericin, Fluconazole, Posaconazole, Voriconazole	>1012 days ^Δ^	Partial improvement	No
10	Meningitis	Coccidioides	Salvage	Amphotericin, Fluconazole, & Voriconazole	201	Resolution	No
11	Meningitis	*Coccidioides immitis*	Salvage	Fluconazole & Voriconazole	>996 days ^Δ^	Partial improvement	No
12	Meningoencephalitis	*Cryptococcus* spp.	Step-down therapy	Amphotericin & Flucytosine	303	Resolution	No
13	Cerebral abscess	*Rhizomucor* spp.	Salvage therapy ^δ^	Amphotericin & Voriconazole	479	Partial improvement	No
14	Cerebral abscess	Unknown	Salvage	Amphotericin, Fluconazole, Posaconazole, & Voriconazole	286	Resolution	No
15	Skull base osteomyelitis	Unknown	Step-down therapy	Amphotericin	510	Resolution	No

* Six weeks from the time of ISA initiation. ^Δ^ Patient on ongoing ISA at end of this review period. ^δ^ In conjunction with ongoing Amphotericin.

## Data Availability

Data available on request only due to privacy and ethical restrictions.

## References

[1] Harrison N., Mitterbauer M., Tobudic S., Kalhs P., Rabitsch W., Greinix H., Burgmann H., Willinger B., Presterl E., Forstner C. (2015). Incidence and characteristics of invasive fungal diseases in allogeneic hematopoietic stem cell transplant recipients: A retrospective cohort study. BMC Infect. Dis..

[2] Biyun L., Yahui H., Yuanfang L., Xifeng G., Dao W. (2024). Risk factors for invasive fungal infections after haematopoietic stem cell transplantation: A systematic review and meta-analysis. Clin. Microbiol. Infect..

[3] Pennington K.M., Martin M.J., Murad M.H., Sanborn D., Saddoughi S.A., Gerberi D., Peters S.G., Razonable R.R., Kennedy C.C. (2024). Risk Factors for Early Fungal Disease in Solid Organ Transplant Recipients: A Systematic Review and Meta-analysis. Transplantation.

[4] Kontoyiannis D.P., Marr K.A., Park B.J., Alexander B.D., Anaissie E.J., Walsh T.J., Ito J., Andes D.R., Baddley J.W., Brown J.M. (2010). Prospective surveillance for invasive fungal infections in hematopoietic stem cell transplant recipients, 2001–2006: Overview of the Transplant-Associated Infection Surveillance Network (TRANSNET) Database. Clin. Infect. Dis..

[5] Pappas P.G., Alexander B.D., Andes D.R., Hadley S., Kauffman C.A., Freifeld A., Anaissie E.J., Brumble L.M., Herwaldt L., Ito J. (2010). Invasive fungal infections among organ transplant recipients: Results of the Transplant-Associated Infection Surveillance Network (TRANSNET). Clin. Infect. Dis..

[6] Kontoyiannis D.P., Patterson T.F. (2014). Diagnosis and treatment of invasive fungal infections in the cancer patient: Recent progress and ongoing questions. Clin. Infect. Dis..

[7] Nivoix Y., Levêque D., Herbrecht R., Koffel J.-C., Beretz L., Ubeaud-Sequier G. (2008). The enzymatic basis of drug-drug interactions with systemic triazole antifungals. Clin. Pharmacokinet..

[8] Wirth F., Ishida K. (2020). Antifungal drugs: An updated review of central nervous system pharmacokinetics. Mycoses.

[9] Lewis J.S., Wiederhold N.P., Hakki M., Thompson G.R. (2022). New Perspectives on Antimicrobial Agents: Isavuconazole. Antimicrob. Agents Chemother..

[10] Patterson T.F., Thompson G.R., Denning D.W., Fishman J.A., Hadley S., Herbrecht R., Kontoyiannis D.P., Marr K.A., Morrison V.A., Nguyen M.H. (2016). Practice Guidelines for the Diagnosis and Management of Aspergillosis: 2016 Update by the Infectious Diseases Society of America. Clin. Infect. Dis..

[11] Miceli M.H., Kauffman C.A. (2015). Isavuconazole: A New Broad-Spectrum Triazole Antifungal Agent. Clin. Infect. Dis..

[12] Ledoux M.-P., Denis J., Nivoix Y., Herbrecht R. (2018). Isavuconazole: A new broad-spectrum azole. Part 2: Pharmacokinetics and clinical activity. J. Mycol. Medicale.

[13] Schmitt-Hoffmann A.-H., Kato K., Townsend R., Potchoiba M.J., Hope W.W., Andes D., Spickermann J., Schneidkraut M.J. (2017). Tissue Distribution and Elimination of Isavuconazole following Single and Repeat Oral-Dose Administration of Isavuconazonium Sulfate to Rats. Antimicrob. Agents Chemother..

[14] Marty F.M., Perfect J.R., Cornely O.A., Mullane K.M., Rahav G., Lee M., Ito M., Maher R., Zeiher B., Ostrosky-Zeichner L. (2014). 824An Open-Label Phase 3 Study of Isavuconazole (VITAL): Focus on Mucormycosis. Open Forum Infect. Dis..

[15] Maertens J.A., Raad I.I., Marr K.A., Patterson T.F., Kontoyiannis D.P., Cornely O.A., Bow E.J., Rahav G., Neofytos D., Aoun M. (2016). Isavuconazole versus voriconazole for primary treatment of invasive mould disease caused by Aspergillus and other filamentous fungi (SECURE): A phase 3, randomised-controlled, non-inferiority trial. Lancet Lond. Engl..

[16] Marty F.M., Ostrosky-Zeichner L., Cornely O.A., Mullane K.M., Perfect J.R., Thompson G.R., Alangaden G.J., Brown J.M., Fredricks D.N., Heinz W.J. (2016). Isavuconazole treatment for mucormycosis: A single-arm open-label trial and case-control analysis. Lancet Infect. Dis..

[17] Jenks J.D., Salzer H.J., Prattes J., Krause R., Buchheidt D., Hoenigl M. (2018). Spotlight on isavuconazole in the treatment of invasive aspergillosis and mucormycosis: Design, development, and place in therapy. Drug Des. Devel. Ther..

[18] Thompson G.R., Rendon A., Ribeiro Dos Santos R., Queiroz-Telles F., Ostrosky-Zeichner L., Azie N., Maher R., Lee M., Kovanda L., Engelhardt M. (2016). Isavuconazole Treatment of Cryptococcosis and Dimorphic Mycoses. Clin. Infect. Dis..

[19] Heidari A., Quinlan M., Benjamin D.J., Laurence B., Mu A., Ngai T., Hoffman W.J., Cohen S.H., McHardy I., Johnson R. (2019). Isavuconazole in the Treatment of Coccidioidal Meningitis. Antimicrob. Agents Chemother..

[20] Schwartz S., Cornely O.A., Hamed K., Marty F.M., Maertens J., Rahav G., Herbrecht R., Heinz W.J. (2020). Isavuconazole for the treatment of patients with invasive fungal diseases involving the central nervous system. Med. Mycol..

[21] Heidari A., Sharma R., Shakir Q., Shah M., Clement J., Donnelley M.A., Reynolds T., Trigg K., Jolliff J., Kuran R. (2023). Isavuconazole in the Treatment of Chronic Forms of Coccidioidomycosis. Clin. Infect. Dis..

[22] Donnelly J.P., Chen S.C., Kauffman C.A., Steinbach W.J., Baddley J.W., Verweij P.E., Clancy C.J., Wingard J.R., Lockhart S.R., Groll A.H. (2020). Revision and Update of the Consensus Definitions of Invasive Fungal Disease From the European Organization for Research and Treatment of Cancer and the Mycoses Study Group Education and Research Consortium. Clin. Infect. Dis..

[23] Ashley E.D. (2019). Antifungal Drugs: Special Problems Treating Central Nervous System Infections. J. Fungi..

[24] Schwartz S., Reisman A., Troke P.F. (2011). The efficacy of voriconazole in the treatment of 192 fungal central nervous system infections: A retrospective analysis. Infection.

[25] Levine M.T., Chandrasekar P.H. (2016). Adverse effects of voriconazole: Over a decade of use. Clin. Transplant..

[26] Fontana L., Perlin D.S., Zhao Y., Noble B.N., Lewis J.S., Strasfeld L., Hakki M. (2020). Isavuconazole Prophylaxis in Patients with Hematologic Malignancies and Hematopoietic Cell Transplant Recipients. Clin. Infect. Dis..

